# Leadership behaviours and health-related early exit from employment: a prospective cohort study of 55 364 employees

**DOI:** 10.1093/eurpub/ckac098

**Published:** 2022-08-25

**Authors:** Kathrine Sørensen, Jeppe Karl Sørensen, Lars L Andersen, Julie Eskildsen Bruun, Paul Maurice Conway, Elisabeth Framke, Ida E H Madsen, Helena Breth Nielsen, Mads Nordentoft, Karina G V Seeberg, Reiner Rugulies

**Affiliations:** National Research Centre for the Working Environment, Copenhagen, Denmark; Department of Psychology, University of Copenhagen, Copenhagen, Denmark; National Research Centre for the Working Environment, Copenhagen, Denmark; National Research Centre for the Working Environment, Copenhagen, Denmark; National Research Centre for the Working Environment, Copenhagen, Denmark; Department of Psychology, University of Copenhagen, Copenhagen, Denmark; National Research Centre for the Working Environment, Copenhagen, Denmark; The Danish Multiple Sclerosis Registry, Copenhagen University Hospital, Copenhagen, Denmark; National Research Centre for the Working Environment, Copenhagen, Denmark; National Research Centre for the Working Environment, Copenhagen, Denmark; National Research Centre for the Working Environment, Copenhagen, Denmark; National Research Centre for the Working Environment, Copenhagen, Denmark; National Research Centre for the Working Environment, Copenhagen, Denmark; Department of Psychology, University of Copenhagen, Copenhagen, Denmark; Department of Public Health, University of Copenhagen, Copenhagen, Denmark

## Abstract

**Background:**

Absence of certain leadership behaviours, such as lack of feedback, recognition and involvement in employee development, has been associated with long-term sickness absence. We tested the hypothesis that absence of eight specific behaviours predicts health-related early exit from employment, and investigated differential effects in subgroups to guide future preventive initiatives.

**Methods:**

Using Cox-proportional hazard modelling, we examined the prospective association between absence of leadership behaviours and health-related early exit from employment in a sample of 55 364 employees during 4.3 years follow-up. Leadership behaviours were measured by employee ratings in national surveys from 2012 to 2016. Exit from employment included disability pension and related measures of health-related early exit, retrieved from a national registry.

**Results:**

We identified 510 cases of health-related early exit from employment during follow-up. A high level of absence of leadership behaviours, was associated with an increased risk of exit from employment (hazard ratio: 1.57, 95% CI: 1.31; 1.89). Subgroup analyses showed that the association between absence of leadership behaviours and exit from employment was similar for women and men and across age groups. The association was stronger for employees with high level of education than for employees with medium/low education, and the association was not observed among employees with a prevalent depressive disorder.

**Conclusions:**

Absence of the eight leadership behaviours is a risk factor for health-related early exit from employment in the Danish workforce. More studies are needed to confirm the results.

## Introduction

Health-related early exit from employment, e.g. disability pensioning, is a major challenge in many countries, especially in those European countries with an ageing society. The old-age dependency rate is increasing, meaning that for every person in the working-age group (15–64 years of age), the number of persons aged 65 or older, who might depend on the working-age population, is rising.^1^ Thus, it has become a major task for public health research to establish risk factors for health-related early exit from employment that are amenable to change, to help identify ways to protect the work-ability of persons in the working-age group.

Knowledge on the role of psychosocial working conditions in relation to early exit from employment is sparse. A recent systematic review of the literature on ‘the contribution of psychological, social and organizational work factors to risk of disability retirement’ concluded that research is mostly limited to a few selected working conditions, in particular shift work and factors related to the ‘job strain model’, i.e. the combination of high job demands with low job control.^2^ Other psychosocial working conditions, including leadership behaviours, have been investigated rarely.

A recent study of the Danish workforce showed that absence of eight specific leadership behaviours, such as involving employees in planning of their own work or providing necessary feedback, was associated with an increased risk of employees’ long-term sickness absence.^3^ One can assume that leadership is also related to exit from employment; however, the evidence about such a relationship is sparse and inconsistent.

Six prospective studies have previously investigated the association between leadership characteristics and disability pension, a key measure of health-related exist from employment. Five studies were conducted with relatively small samples (967–6748 participants) and provided inconsistent results, with two studies reporting a significant association^4^^,^^5^ and three reporting no associations.^6–8^ These inconsistent results may be due to lack of statistical power, as early exit from employment is a relatively rare outcome, requiring large sample sizes for calculating estimates with acceptable precision. The only large-scale study (40 554 participants) reported no association between leadership quality and risk of disability pension,^9^ however, the measurement of leadership was limited to a four-item scale.

The aim of this study was therefore to examine the association between leadership behaviours and risk of health-related early exit from employment, including but not limited to disability pension, in a large-scale prospective study of the Danish workforce. The large study sample allowed us to estimate the association between leadership behaviour and work exit with a high level of precision and to conduct analyses in subgroups. Further, we were able to measure leadership comprehensively, assessing leadership behaviours by means of eight specific leader behaviours that we deemed as supportive or beneficial for the employees. We hypothesized that the absence of these behaviours would be associated with an increased risk of health-related early exit from employment. In addition to this hypothesis, we also explored whether associations between leadership behaviours and risk of health-related exit from employment differ in subgroups defined by sex, age, educational level and prevalence of depressive disorders at baseline.

## Methods

### Study design and participants

We conducted a prospective cohort study by merging data on leadership behaviours from the nationwide questionnaire-based Work Environment and Health in Denmark (WEHD) survey with national register data on social transfer payments from the Danish Register for Evaluation of Marginalization (DREAM).^10^^,^^11^ A detailed description of WEHD is published elsewhere.^12^

Respondents of the WEHD waves from 2012, 2014 and 2016 were initially selected (*n* = 67 407); of these, 62 289 were working at baseline. If a participant responded to more than one wave of WEHD, the first response was chosen. We pre-censored 2775 respondents, because they emigrated (*n* = 489), retired (*n* = 311) or registered with health-related early exit from employment (*n* = 1623) before the start of the follow-up, or were censored between filling in the survey and 1 January 2013 (*n* = 341, for the 2012 wave only). Respondents from the 2014 and 2016 waves were followed from the day they filled in the questionnaire until 31 January 2019. Respondents from the 2012 wave were followed not from the day they filled in the questionnaire but from 1 January 2013, because on this day a major political reform of the Danish disability pension system came into force^13^ and we wanted to ensure that the same disability pension policies applied to all participants throughout the follow-up period.

We further excluded 1717 participants who had no leader, and lastly we excluded participants with missing values on key variables including sex, age, educational level, depressive state and sample type (*n* = 2433). The final population consisted of 55 364 participants (see flowchart, Supplementary figure SA1).

### Health-related early exit from employment

Data on health-related early exit from employment were retrieved from DREAM.^10^^,^^11^ Health-related early exit from employment was assessed using the DREAM codes for (i) disability pension, (ii) disability pension while working in a light job with limited work demands, (iii) enrolment in a work-ability assessment program (a prerequisite of disability pension) or (iv) starting in a special protected employment scheme for workers with severe health problems.

### Leadership behaviour

Leadership behaviours were measured in WEHD by asking the respondents to rate how often eight specified behaviours of their closest leader or the management occurred. See textbox 1 for a list of included items. The response categories ranged from ‘Always’ (scored as one) to ‘Never’ (scored as five) and were added together to form a total score. A higher score on the scale thus indicates more absence of the leadership behaviours considered.

When values were missing on <4 items, the sum score was calculated by replacing the missing items with the average of the non-missing items. We excluded respondents with missing values on more than four out of the eight items.

We operationalized leadership behaviour as a continuous variable (one-point increase of the score on the scale), as well as a categorical variable with four categories based on quartiles of the distribution, which allowed us to investigate a dose–response relationship. Furthermore, we made the *post-hoc* decision to also include a dichotomous variable. The dichotomous variable was constructed by collapsing the first three quartiles of the leadership behaviours score (low, medium low and medium high absence) into one group and compare this group with the fourth quartile (high absence of the leadership behaviours). A more detailed description and validation of the leadership behaviour scale is provided in the Supplementary material.

### Covariates

As covariates, we included sex, age, educational level, sampling method, presence of a depressive disorder at baseline and eligibility for disability pension (i.e. age ≥40 years, as younger individuals usually do not get granted a disability pension in Denmark). Sex, age and educational level were retrieved from population registers.^14^^,^^15^ Highest educational attained was categorized into low (less than high school and no vocational education), medium (high school degree or completed vocational education) and high (post-secondary education and above). As the population in WEHD was sampled through two different methods,^12^ we included a variable that indicates sampling method.

Presence of a depressive disorder at baseline was ascertained with the Major Depression Inventory (MDI), a comprehensively validated self-administered rating scale.^16–18^ In accordance with recommendations in the literature, an MDI-score of ≥21 points was used to indicate a probable depressive disorder.^17^

For the sensitivity analyses, we included additional covariates from registers job type and occupational industry, both based on standard classifications from register data,^19^^,^^20^ immigration status, organization type (private vs. public), and also included self-reported seniority of the participant from WEHD.

### Statistical analysis

Using Cox-proportional hazard models with weeks since baseline as the underlying time scale, we tested the prospective association between absence of the leadership behaviours and subsequent risk of health-related early exit from employment. Participants were followed until health-related early exit from employment or censored due to non-health based retirement, emigration, death or end of follow-up (31 January 2019), whichever of these occurred first.

We analyzed the association between absence of leadership behaviour and risk of health-related early exit from employment for the continuous, four-category and dichotomous measure of leadership behaviours. We calculated crude associations and associations adjusted for age, sex, educational level, type of sample and eligibility for disability pension (Model 1), and additionally for depressive disorder at baseline (Model 2). Further, we tested the associations within the above-mentioned subgroups and for the interaction between subgroup and category of leadership behaviour, defined as deviation from multiplicativity.

Finally, we conducted a number of sensitivity analyses; one with a restricted outcome that only included disability pension, a series where we adjusted for wave of WEHD, job type and industry, immigrant status, seniority of the employee, organization type and lastly by analyzing each of the eight items of the leadership scale separately.

## Results

Table 1 shows the characteristics of the study population and the number of cases per 10 000 person-years. There were slightly more women (53.1%) than men (46.9%), the mean age was 45.2 years (standard deviation: 11.2 years), and most participants had a middle (44.0%) or high (42.9%) level of education. There were 4168 participants (7.5%) with an indication of a depressive disorder.

**Table 1:** Study population characteristics and incidence of health-related early exit from employment

	*n*	% of population	Cases	Person-years	Cases per 10 000 person years
Total sample	55 364	100.0	510	236 989	21.5
Sex					
Women	29 412	53.1	310	127 101	24.4
Men	25 952	46.9	200	109 887	18.2
Age categories					
18–29	6129	11.1	20	27 251	7.3
30–49	26 621	48.1	219	121 898	18.0
≥50	22 614	40.8	271	87 840	30.9
Educational level					
Low	7266	13.1	109	30 356	35.9
Middle	24 353	44.0	246	104 236	23.6
High	23 745	42.9	155	102 396	15.1
Depressive disorder at baseline					
Yes	4168	7.5	119	17 534	67.9
No	51 196	92.5	391	219 455	17.8
Job type^a^					
1: Managers	2590	4.7	15	10 978	13.7
2: Professionals	17 670	31.9	120	76 679	15.6
3: Technicians and associate professionals	7674	13.9	61	32 898	18.5
4: Clerical support workers	5000	9.0	41	21 024	19.5
5: Services and sales workers	8380	15.1	119	36 589	32.5
6: Skilled agricultural, forestry and fishery workers	338	0.6	<5^b^	1394	–
7: Craft and related trade workers	4609	8.3	34	19 565	17.4
8: Plant and machine operators and assemblers	3290	5.9	49	13 171	37.2
9: Elementary occupations	5488	9.9	66	23 186	28.5
0: Armed forces occupations	325	0.6	<5^b^	1504	–
Missing	0	–	–	–	–
Industry^a^					
1: Agriculture, forestry and fishing	583	–	<5^b^	2357	–
2: Manufacturing, mining and quarrying and utility services	9059	16.4	83	37 304	22.2
3: Construction	3022	5.5	33	13 357	24.7
4: Trade and transport etc.	9244	16.7	68	39 542	17.2
5: Information and communication	1940	3.5	6	8306	7.2
6: Financial and insurance	1955	3.5	12	8739	13.7
7: Real estate	562	1.0	9	2388	37.7
8: Other business services	4849	8.8	45	20 864	21.6
9: Public administration, education and health	22 234	40.2	231	96 317	24.0
10: Arts, entertainment and other services	1909	3.4	20	7785	25.7
Missing	7	–	<5^b^	30	–
Type of organisation^a^					
Private	31 531	57.0	269	134 276	20.0
Public	23 831	43.0	241	102 707	23.5
Immigration status^a^					
Danish	52 102	94.1	473	223 333	21.2
Immigrant or descendent of immigrant	3234	5.8	37	13 643	27.1
Seniority at baseline^a^					
≤3 months	1392	2.5	12	6086	19.7
3 months to 1 year	4849	8.8	55	20 959	26.2
1–3 years	8873	16.0	78	38 546	20.2
3–5 years	7331	13.2	59	33 079	17.8
5–10 years	11 784	21.3	109	50 861	21.4
>10 years	21 073	38.1	195	87 150	22.4

a Covariates are only used in sensitivity analyses shown in Supplementary material.

b Due to protection of the individual participants data, number of cases below five cannot be shown for this specific category.

### Leadership behaviour and risk of health-related early exit from employment

During a mean follow-up time of 4.28 (SD 1.8) years, we identified 510 cases of health-related early exit from employment (21.5 per 10 000 person-years) (table 1). Of the 510 cases, 179, 67 and 264 were due to disability pension, enrolment in a work-ability assessment program and special protected employment scheme for workers with severe health problems, respectively.

Number of cases per 10 000 person-years was higher in women than men, higher in participants of older age than participants of younger age, higher in participants with a lower education level than participants of higher education and higher in participants with a depressive disorder than in participants without a depressive disorder.

The risk of health-related early exit from employment in relation to absence of the leadership behaviours is presented in table 2. A one-point increase on the sum score was associated with a higher risk of health-related early exit from employment [hazard ratio (HR): 1.03, 95% CI: 1.01; 1.04], after adjustment for age, sex, educational level, type of sample and eligibility for disability pension (Model 1). After further adjusting for depressive disorder at baseline, the association became statistically non-significant (HR = 1.01, 95% CI: 0.99; 1.02, Model 2).

**Table 2:** Association between absence of the leadership behaviours and health-related early exit from employment

					Crude		Model 1^a^		Model 2^b^	
	*n*	Person-years	Cases	Cases pr. 10 000 person years	HR	(95% CI)	HR	(95% CI)	HR	(95% CI)
Absence of leadership behaviour										
Per 1 increase on sum score^c^	55 364	236 989	510	21.5	1.03	(1.01; 1.04)	1.03	(1.01; 1.04)	1.01	(0.99; 1.02)
Absence of the leadership behaviours, in quartiles										
Low	15 062	63 975	125	19.5	1	ref.	1	ref.	1	ref.
Medium low	11 980	51 894	87	16.8	0.86	(0.65; 1.13)	0.89	(0.68; 1.18)	0.86	(0.65; 1.13)
Medium high	14 607	63 080	122	19.3	0.99	(0.77; 1.28)	1.02	(0.79; 1.31)	0.93	(0.72; 1.19)
High	13 715	58 040	176	30.3	1.55	(1.24; 1.96)	1.53	(1.22; 1.93)	1.16	(0.92; 1.48)
Absence of the leadership behaviours, dichotomized										
Low/medium low/medium high	41 649	178 949	334	18.7	1	ref.	1	ref.	1	ref.
High	13 715	58 040	176	30.3	1.62	(1.35; 1.95)	1.57	(1.31; 1.89)	1.25	(1.03; 1.51)

a Model 1: adjusted for sex, age, educational level, type of sample and eligibility for disability pension (age 40 or above).

b Model 2: as Model 1 and further adjusted for depressive disorder at baseline.

c High sum score is equivalent to a high degree of absence of leadership behaviour.

Using the quartiles of the sum score, we found a higher risk of health-related early exit from employment among individuals in the high-quartile group (indicating absence of leadership behaviours) when compared with those in the low-quartile group, with a HR of 1.57 (95% CI: 1.22; 1.39, Model 1). After further adjustment for depressive disorder (Model 2), the estimate became statistically non-significant (HR = 1.16; 95% CI: 0.92; 1.48). We found no increased risk among individuals in the second and third quartile of the sum score.

Using the dichotomized sum score, we found an increased risk of health-related early exit from employment among individuals in the fourth quartile with an absence of the leadership behaviours compared to individuals in the other quartile (first, second and third quartiles combined), with a HR of 1.57 (95% CI: 1.31; 1.89) in Model 1 and an HR of 1.25 (95% CI: 1.03; 1.51) when adjusting for depressive disorder in Model 2.

The sensitivity analyses yielded results similar to the main analysis (Supplementary table SA1). The results of the sensitivity analyses considering each of the eight leadership behaviour item analyzed separately, are presented in Supplementary table SA2. The analysis showed that all items presented statistically significant associations with early exit from employment in Model 1.

### Subgroup analyses

Table 3 shows the association between the dichotomized exposure and risk of health-related early exit from employment in the subgroups defined by sex, age, education and depressive disorder at baseline, including tests for interaction. We found no effect modification, measured by multiplicative interaction, in terms of sex or the age groups (below or above 50 years of age). The association between the leadership behaviours and health-related early exit from employment was stronger in those with a level of high education than in those with a low or medium level of education (*P* for interaction 0.06 and 0.07, respectively). Among those without depressive disorder, there was a statistically significant association between absence of the leadership behaviours and risk of health-related early exit from employment (HR: 1.49; 95% CI: 1.21; 1.85), whereas there was no statistically significant association among those with depressive disorder (HR: 0.77; 95% CI: 0.53; 1.10) (*P* < 0.01 for interaction).

**Table 3:** Association between absence of the leadership behaviours (dichotomized) and health-related early exit from employment in subgroups

					Model 1^a^			Model 2^b^		
	*n*	Person-years	Cases	Cases per 10 000 person-years	HR	(95% CI)	*P*-value on interaction term	HR	(95% CI)	*P*-value on interaction term
By sex							0.48			0.28
Women										
Low/medium low/medium high	22 371	96 873	210	21.7	1	ref.		1	ref.	
High	7041	30 228	100	33.1	1.49	(1.17; 1.89)		1.18	(0.92; 1.52)	
Men										
Low/medium low/medium high	19 278	82 076	124	15.1	1	ref.		1	ref.	
High	6674	27 812	76	27.3	1.70	(1.27; 2.26)		1.35	(1.01; 1.82)	
By age							0.28			0.26
Age <50										
Low/medium low/medium high	24 789	113 149	163	14.4	1	ref.		1	ref.	
High	7961	35 999	76	21.1	1.41	(1.07; 1.85)		1.16	(0.87; 1.53)	
Age ≥50										
Low/medium low/medium high	16 860	65 800	171	26.0	1	ref.		1	ref.	
High	5754	22 040	100	45.4	1.74	(1.36; 2.22)		1.37	(1.06; 1.78)	
By educational level							0.06			0.07
Low and medium educational level										
Low/medium low/medium high	23 288	99 395	237	23.8	1	ref.		1	ref.	
High	8331	35 198	118	33.5	1.41	(1.13; 1.76)		1.14	(0.91; 1.44)	
High educational level										
Low/medium low/medium high	18 361	79 554	97	12.2	1	ref.		1	ref.	
High	5384	22 842	58	25.4	2.03	(1.47; 2.81)		1.53	(1.09; 2.15)	
By depressive disorder							<0.01			
No depressive disorder										
Low/medium low/medium high	39 624	170 350	270	15.8	1	ref.				
High	11 572	49 104	121	24.6	1.49	(1.21; 1.85)				
High depressive disorder										
Low/medium low/medium high	2025	8599	64	74.4	1	ref.				
High	2143	8935	55	61.6	0.77	(0.53; 1.1)				

a Model 1 is adjusted for sex, age, educational level, type of sample and eligibility for disability pension (age 40 or above) except the variable used for stratification.

b Model 2 like Model 1 and further adjusted for depressive disorder.

## Discussion

In this nationwide, prospective cohort study in Denmark, we found that employees reporting an absence of eight leadership behaviours from their supervisors were at increased risk of health-related early exit from employment. The association was similar in women and men and in those of younger and older age, but tended to be stronger among those with a higher level of education than among those with low or medium level of education. Participants with a prevalent depressive disorder at baseline had, as expected, a higher risk of health-related early exit from employment. However, in this subgroup, absence of the leadership behaviours did not appear to further increase the risk of health-related early exit from employment.

In the study, we treated the sum score of leadership behaviours in three different ways; as continuous score, categorized into quartiles and dichotomized (highest quartile as indicator of exposure vs. the lower quartiles). All three operationalizations showed that higher absence of the leadership behaviours was associated with an increased risk of early exit from employment before adjustment for prevalent depressive disorder at baseline. After adjustment for prevalent depressive disorder at baseline, the estimates attenuated and statistical significance was lost for the continuous score and the score categorized in quartiles, whereas the dichotomized score retained a statistical significant association with risk of health-related exit from employment. This suggests that the group that bears the risk is mainly the ‘extreme’ group, wherein the leadership behaviours is absent to a large extent.

Other studies have investigated the association between leadership and the risk of disability pension. Our result is different from the only large-scale study (40 554 participants) that found no association between a four-item leadership quality scale and the risk of disability pension.^9^

The stratum consisting of individuals with depressive disorder contained 4168 (7.5%) of the participants; however, the group also contained 119 of 510 cases (23.3%). We assumed that depression would be a confounder due to this strong association between depressive disorder at baseline and later early exit from employment (table 1) and the possible association between depressive disorder and the reporting of absence of leadership behaviours. Indeed, the analyses showed that adjusting for baseline depressive disorder strongly attenuated the association between absence of the leadership behaviours and risk of health-related exit from employment (table 2). However, when we stratified by depressive disorder, instead of adjusting for depressive disorder (table 3), we found that the HR for the association between absence of leadership behaviours and risk of health-related early exit from employment was 1.49 among those without a baseline depressive disorder and 0.77 among those with a baseline depressive disorder. A multiplicative interaction analyses showed that this difference was statistically significant. It is notable that we found that the association is not only different in the two strata, but actually points into different directions.

In the stratum consisting of individuals with depressive disorder at baseline a possible explanation for the lack of an association between absence of the leadership behaviours and increased risk of health-related early exist of employment could be that individuals with a depressive disorder are already at a high risk for health-related early exit from employment and this pull away from employment may be so strong that absence of the leadership behaviours that we investigate in this study play an insubstantial role for this group.

Overall, the results suggest that baseline depressive disorder may act more as an effect modifier than a confounder. However, it cannot be ruled out that, in some cases, absence of the leadership behaviours may have contributed to the onset of a depressive disorder, meaning that a depressive disorder may, at least partly, operate also as a mediator for the association between absence of the leadership behaviours and risk of health-related early exit from employment. Further studies with repeated measures of both leadership behaviour and depressive disorder are needed to examine to what extent a depressive disorder is a confounder, an effect modifier or a mediator in the association between absence of leadership behaviour and risk of health-related early exit from employment.

Our results suggest level of education as a further effect modifier, as the association between absence of the leadership behaviours and risk of health-related early exit from employment was stronger among participants with a high level of education than among participants with a low or medium level of education. This is in agreement with a previous Danish study showing that high workplace social capital, a construct that includes measures of leadership behaviour, was more strongly associated with a decreased risk of long-term sickness absence among employees of high occupational grade than among employees of low occupational grade.^21^ A possible explanation could be that a higher level of complexity in jobs requiring a high level of education also makes the presence of leadership behaviours of supervisors more important.

### Strengths and limitations

A strength of the study is the size of the study population, providing the necessary statistical power for a detailed analysis of both main associations and interactions. Furthermore, the study employs a longitudinal design, with exposure (self-reported) and outcome (register-based) variables measured by two different methods, thereby reducing the risk of common method variance.

A limitation of this study is that we only measured absence of the leadership behaviours at one point in time, instead of using repeated measures, and that we measured absence of the leadership behaviour and depressive disorder with self-reported questionnaires, which may have led to misclassification. Possible fluctuations in the rating of leadership behaviours during follow-up are therefore not accounted for in this study. Leadership behaviour was rated by the employee, meaning that our measure captured the individual perception of absence of leadership behaviour. Therefore, we lack information on leader behaviours as observed by a third-party observer or on the leaders’ perception of their own behaviours. Further, we do not know the reasons why leaders displayed an absence of the leadership behaviours. It is possible that some leaders lacked leadership skills and therefore their leadership behaviours were absent. It is also possible that absence of leadership behaviours was not due to lack of leadership skills but to lack of resources in the organization, which may also affect the work environment as a whole, for instance in terms of high job demands and low control. Some leaders may have been overwhelmed with other work tasks and consequently did not have the time resources to give feedback, recognition and support to the employees.

Whether different reasons for absence of the leadership behaviours could have a different impact on risk of health-related early exit from employment should be examined in further studies.

Another limitation of the study is that we did not include other work environment factors of the employee, or other indicators of the relationship between the leader/management and the employee.

### Practical implication

Our results suggest providing a higher amount of the investigated leadership behaviours may contribute to the prevention of health-related early exit from employment. As this is an observational study, such a conclusion needs to be verified in workplace intervention studies. We did not find evidence that absence of leadership behaviour increased the risk of health-related early exit from employment for employees with a depressive disorder in our study, suggesting that an intervention based on the leadership behaviours that we investigated here might not be beneficial in terms of reducing early exit from employment among this group.

## Conclusion

In conclusion, this study suggests that absence of leadership behaviours is a risk factor of health-related early exit from employment in the Danish workforce. The risk was similar for women and men and for younger and older employees, while it was somewhat higher for employees with a high level of education than for employees with a medium or low level of education. Employees with a prevalent depressive disorder had a higher risk of health-related early exit from employment, but a higher level of absence of leadership behaviours was not associated with a further increase of the risk of health-related early exit from employment. More studies are needed to confirm the results, e.g. studies with more comprehensive measures of leadership behaviours.

## Supplementary data

Supplementary data are available at *EURPUB* online.

## Funding

The study was supported by grants from the Danish Working Environment Research Fund (grant numbers: 10-2016-03, 01-2018-03 and 10-2019-03). The funder had no further role in the study design, the collection, analyses and interpretation of data; the writing of the manuscript; or the decision to submit the manuscript for publication.

##  

*Conflicts of interest*: None declared.

Key points

- In this prospective study in the Danish workforce, absence of specific leadership behaviours predicted an increased risk of health-related early exit from employment.
- In a subgroup of employees with prevalent depressive disorder at baseline, which had a markedly increased risk of health-related early exit from employment, absence of the leadership behaviours did not further increase the risk.
- Whether facilitating more of the leadership behaviours may reduce risk of health-related early exit from employment should be investigated in workplace intervention studies.

Textbox 1 The eight items used to measure leadership behaviour

Each item could be answered with: ‘1 = Always’, ‘2 = Often’, ‘3 = Sometimes’, ‘4 = Seldom’, ‘5 = Never’ or ‘Have no leader’ (individuals with no leader were excluded from the study).

How often -

- L1: does your immediate leader explain the company’s objectives so you understand what they mean for your assignments?
- L2: do you have sufficient authority in relation to your responsibility?
- L3: does your immediate leader take the time to engage in your professional development?
- L4: does your immediate leader involve you in the planning of your work?
- L5: does your immediate leader give you the necessary feedback (favourable and critical)?
- L6: is your work recognized and appreciated by the management?
- L7: do you get the necessary help and support from your immediate leader?
- L8: can you rely on announcements from the management?

## Supplementary Material

ckac098_Supplementary_Data
