# Supplementary material for: Leadership behaviours and health-related early exit from employment: a prospective cohort study of 55 364 employees
Source: Eur J Public Health. 2022 Aug 25;32(5):709–15. doi: 10.1093/eurpub/ckac098 (PMC9527957; doi:10.1093/eurpub/ckac098)
Supplement: ckac098_Supplementary_Data [file ckac098_supplementary_data.docx]

**Supplementary Material**

**Figure A1**

Flowchart

First time respondents from Work environment and health survey 2012, 2014 and 2016
n=67,407

Working at baseline
n= 62,289

No precensoring
n= 59,514

Have leader
n= 57,797

Not working or information missing on working status (n=5,118)

Precensored, due to emigration (n=489), retirement (n=311), registration with health-related early exit from employment in the two years before baseline (n=1,623) or censoring or health-related early exit between filling in the survey and January 1st 2013 (n=341, for the 2012 wave only)

n=2,850

Have no leader
(n=1,717)

Missing on key variables:
Sex, age, educational level, depressive state and sample type (n=2,433)

Final population
n= 55,364

Final population

n= 55,271

**Table A1**

*Sensitivity analysis with (i) disability pension only, (ii) adjustment for sample year, (iii) adjustment for job type and industry, (iv) adjustment for immigrant status, (v) seniority of employee, and (vi) organisation type (public or private)*

|  |  | Model 1* | | |  | Model 2** | | |
| --- | --- | --- | --- | --- | --- | --- | --- | --- |
|  |  | HR |  | (95% CI) |  | HR |  | (95% CI) |
| Absence of leadership behavior, continous |  |  |  |  |  |  |  |  |
| Estimate is increased risk per 1 increase on rating scale | |  |  |  |  |  |  |  |
|  |  |  |  |  |  |  |  |  |
| Main analysis |  | 1.03 |  | (1.01;1.04) |  | 1.01 |  | (0.99;1.02) |
| i) Outcome is restricted to disability pension |  | 1.04 |  | (1.01;1.06) |  | 1.02 |  | (0.99;1.04) |
| ii) Adjusted for sample year |  | 1.03 |  | (1.01;1.04) |  | 1.01 |  | (0.99;1.02) |
| iii) Adjusted for job type and sector |  | 1.02 |  | (1.01;1.04) |  | 1.01 |  | (0.99;1.02) |
| iv) Adjusted for immigrant status |  | 1.03 |  | (1.01;1.04) |  | 1.01 |  | (1.00;1.02) |
| v) Adjusted for seniority of employee |  | 1.03 |  | (1.01;1.04) |  | 1.01 |  | (1.00;1.02) |
| vi) Adjusted for organisation type |  | 1.03 |  | (1.01;1.04) |  | 1.01 |  | (0.99;1.02) |
|  |  |  |  |  |  |  |  |  |
|  |  |  |  |  |  |  |  |  |
|  |  | Model 1* | | |  | Model 2** | | |
|  |  | HR |  | (95% CI) |  | HR |  | (95% CI) |
| Absence of leadership behavior, dichotomized |  |  |  |  |  |  |  |  |
| Low absence of leadership behavior is compared to low/medium low/medium high |  |  |  |  |  |  |  |  |
|  |  |  |  |  |  |  |  |  |
| Main analysis |  | 1.57 |  | (1.31;1.89) |  | 1.25 |  | (1.03;1.51) |
| i) Outcome is restricted to disability pension |  | 1.77 |  | (1.33;2.34) |  | 1.39 |  | (1.04;1.87) |
| ii) Adjusted for sample year |  | 1.59 |  | (1.32;1.90) |  | 1.26 |  | (1.04;1.53) |
| iii) Adjusted for job type and sector |  | 1.53 |  | (1.27;1.84) |  | 1.22 |  | (1.01;1.48) |
| iv) Adjusted for immigrant status |  | 1.57 |  | (1.31;1.89) |  | 1.25 |  | (1.03;1.51) |
| v) Adjusted for seniority of employee |  | 1.58 |  | (1.31;1.89) |  | 1.26 |  | (1.04;1.52) |
| vi) Adjusted for organisation type |  | 1.57 |  | (1.30;1.88) |  | 1.25 |  | (1.03;1.51) |

*Model 1: Adjusted for age, sex, educational level, type of sample and eligibility for disability pension (age 40 or above)

**Model 2: like model 1, and further adjusted for depressive disorder

**Table A2**

*Sensitivity analysis: Separate analysis with every item from the scale (here standardized for better comparison), in a sample of54.979 employees without imputed values on any of the items.*

|  |  | Model 1* | | |  | Model 2** | | |
| --- | --- | --- | --- | --- | --- | --- | --- | --- |
|  |  | HR |  | (95% CI) |  | HR |  | (95% CI) |
| Absence of leadership behaviors |  |  |  |  |  |  |  |  |
| per 1 increase on standardized rating scale |  | 1.2 |  | (1.00;1.19) |  | 1.07 |  | (0.98;1.16) |
|  |  |  |  |  |  |  |  |  |
| Item |  |  |  |  |  |  |  |  |
| L1 |  | 1.09 |  | (1.00;1.19) |  | 1.02 |  | (0.93;1.11) |
| L2 |  | 1.19 |  | (1.09;1.29) |  | 1.07 |  | (0.99;1.17) |
| L3 |  | 1.10 |  | (1.01;1.21) |  | 1.01 |  | (0.92;1.10) |
| L4 |  | 1.09 |  | (1.00;1.19) |  | 1.03 |  | (0.95;1.13) |
| L5 |  | 1.13 |  | (1.04;1.24) |  | 1.04 |  | (0.95;1.14) |
| L6 |  | 1.19 |  | (1.09;1.29) |  | 1.07 |  | (0.98;1.17) |
| L7 |  | 1.19 |  | (1.09;1.30) |  | 1.07 |  | (0.98;1.17) |
| L8 |  | 1.20 |  | (1.11;1.31) |  | 1.08 |  | (0.99;1.18) |

*Model 1: Adjusted for age, sex, educational level, type of sample and eligibility for disability pension (age 40 or above)

**Model 2: like model 1, and further adjusted for depressive disorder

**Scale validation**

The sum score measure of leadership behaviour is a formative measure. In a formative measure, items uniquely contribute to an index, and the content of the index is defined by the items themselves, as opposed to a reflective measure, where an underlying latent factor is expressed through different items (1). We tested the validation of leadership behaviour as a formative measure using two tests among the 54,979 employees who had no imputed values on the eight items (2). First, using the variance inflation factor (VIF) we found an acceptable low level of multi-collinearity between the items, as the maximum VIF for the eight items was 2.9 which is below the suggested limit of 3.3 (3). Second, a multiple indicators and multiple causes (MIMIC) model with the two outcomes “How often did you in the last month feel that difficulties at work were piling up so high that you could not overcome them?” and “To what extent does your work give you confidence and joy of work?” showed an acceptable fit (p(chi-sq)<.001, SRMR= 0.04, CFI=1.00). These two outcomes were chosen because they were job and work environment related outcomes available in WEHD that we expected to be affected by the leadership behaviours (2).

Two items of the leadership behaviour scale were statistically not significant in the MIMIC-model (L1 and L5). According to Roberts and Thatcher, it is acceptable to keep items that are statistically not significant in the MIMIC model in the scale (2), in particular if removing the items from the sum score would mean that one also remove the specific aspects of the construct that the items contribute with. We decided to keep the two items in the scale, because (i) the overall MIMIC-model had acceptable fit, (ii) removing the items would had removed important specific aspects from the leadership behaviour sum score and (iii) we wanted to work with an identical scale that has been used in previous publications from WEHD (4, 5).

To investigate if the measure is stable over time, we calculated correlation coefficients for the leadership behaviour rating in 2012, 2014 and 2016 among a subsample of 6,498 participants who participated in all three rounds of WEHD. The correlation between leadership ratings was 0.56 in 2012 and 2014and 0.46 in 2012 and 2016. We therefore conclude that the scale of leadership behaviours shows an acceptable level of stability across time

1. van Amelsvoort LG, Fleuren BP, Kant IJJoce. Improving measurement models in clinical epidemiology: time to move beyond the inherent assumption of an underlying reflective measurement model. 2020;118:119-23.

2. Roberts N, Thatcher JB. Conceptualizing and Testing Formative Constructs: Tutorial and Annotated Example. Data Base Adv Inf Sy. 2009;40(3):9-39.

3. Diamantopoulos A, Winklhofer HMJJomr. Index construction with formative indicators: An alternative to scale development. 2001;38(2):269-77.

4. Sørensen JK, Framke E, Clausen T, Garde AH, Johnsen NF, Kristiansen J, et al. Leadership Quality and Risk of Long-term Sickness Absence Among 53,157 Employees of the Danish Workforce. Journal of occupational and environmental medicine. 2020;62(8):557-65.

5. Rugulies R, Sørensen JK, Madsen IE, Nordentoft M, Sørensen K, Framke EJEJoPH. Can leadership quality buffer the association between emotionally demanding work and risk of long-term sickness absence? 2021.
